# Oxidative Stress and Diseases Associated with High-Altitude Exposure

**DOI:** 10.3390/antiox11020267

**Published:** 2022-01-28

**Authors:** Eduardo Pena, Samia El Alam, Patricia Siques, Julio Brito

**Affiliations:** Institute of Health Studies, Arturo Prat University, Iquique 1100000, Chile; eduardopena@unap.cl (E.P.); psiques@tie.cl (P.S.); jbritor@tie.cl (J.B.)

**Keywords:** oxidative stress, high altitude, pulmonary hypertension, reactive oxygen species, altitude diseases

## Abstract

Several diseases associated with high-altitude exposure affect unacclimated individuals. These diseases include acute mountain sickness (AMS), high-altitude cerebral edema (HACE), high-altitude pulmonary edema (HAPE), chronic mountain sickness (CMS), and, notably, high-altitude pulmonary hypertension (HAPH), which can eventually lead to right ventricle hypertrophy and heart failure. The development of these pathologies involves different molecules and molecular pathways that might be related to oxidative stress. Studies have shown that acute, intermittent, and chronic exposure to hypobaric hypoxia induce oxidative stress, causing alterations to molecular pathways and cellular components (lipids, proteins, and DNA). Therefore, the aim of this review is to discuss the oxidative molecules and pathways involved in the development of high-altitude diseases. In summary, all high-altitude pathologies are related to oxidative stress, as indicated by increases in the malondialdehyde (MDA) biomarker and decreases in superoxide dismutase (SOD) and glutathione peroxidase (GPx) antioxidant activity. In addition, in CMS, the levels of 8-iso-PGF2α and H_2_O_2_ are increased, and evidence strongly indicates an increase in Nox4 activity in HAPH. Therefore, antioxidant treatments seem to be a promising approach to mitigating high-altitude pathologies.

## 1. Introduction

In recent years, the number of people with acute, intermittent (shift work; days at high altitude and then rest days at sea level, for years), or permanent exposure to high altitudes has increased considerably, and it has been estimated that more than 81.6 million people live at altitudes ≥2500 m [1]. Exposure to high altitude is characterized as a hypobaric hypoxic condition due to the decrease in partial oxygen pressure, which reduces the bioavailability of oxygen in organisms [2]. Hypobaric hypoxia causes changes in the oxygen transport cascade from the air to the mitochondrial membrane [3], which trigger compensatory physiological mechanisms to help the individual tolerate and acclimatize to hypobaric hypoxia. However, in some cases, the individual is susceptible to diseases, of which acute mountain sickness (AMS) is the most common, followed by high-altitude pulmonary edema (HAPE), high-altitude cerebral edema (HACE) [4], high-altitude pulmonary hypertension (HAPH) [5,6], and chronic mountain sickness (CMS) [5,7,8].

Previous studies have shown that both acute and chronic exposure to hypobaric hypoxia increase the levels of oxidative stress biomarkers [9], highlighting that in certain tissues, such as the lung, oxidative stress is rapidly triggered under high-altitude exposure. For example, a study has shown that within 1.5 h of exposure to acute hypobaric hypoxia, the levels of reactive oxygen species (ROS) and the oxidative stress biomarker malondialdehyde (MDA), a product of lipid peroxidation, increase in the lungs of rats [10]. Notably, oxidative stress induced by hypobaric hypoxia causes structural alterations in lipids, proteins, and DNA, resulting in cellular damage [11]. Moreover, several studies have implicated oxidative stress in the development of pathologies induced by hypobaric hypoxia exposure, such as those mentioned above [8,10,12,13,14]. Therefore, the aim of this review is to discuss the oxidative molecules and pathways involved in the development of acute and chronic mountain sickness, pulmonary and cerebral high-altitude edema, and pulmonary hypertension, because this information is necessary to understand and potentially mitigate these pathologies, specifically by revealing pharmacological targets.

## 2. Oxidative Stress under Hypobaric Hypoxia

Reactive oxygen species are small and unstable molecules produced from oxygen (O_2_). These molecules can be generated through different pathways, such as the mitochondrial electron transport chain, uncoupled nitric oxide synthase (NOS), the NADPH oxidase complex, and xanthine oxidase [15]. The principal ROS molecules studied to date are superoxide (O_2_•^−^), hydroxyl radicals (•OH), peroxide radicals (ROO•), the alkoxy group (RO•), hydrogen peroxide (H_2_O_2_), hypochlorous acid (HOCl), ozone (O_3_), singlet oxygen (^1^O_2_), and peroxynitrite (ONOO^−^) [16].

ROS have been related to several physiological processes involved in cellular regulation, such as cell differentiation, apoptosis, and growth, as well as in the immune system [17,18], emphasizing that ROS are now also recognized as important signaling mediators and second messengers that enhance the vasoreactivity of the pulmonary artery [19,20,21]. However, when the levels of ROS are increased through environmental and chemical stimuli and are not compensated by endogenous antioxidant systems, such as superoxide dismutase (SOD), catalase (CAT), glutathione peroxidase/reductase (GSH-Px), peroxiredoxin/thioredoxin (PRX/Trx), or metalloproteases (MMPs), oxidative stress occurs [15,22]. Oxidative stress can alter several cellular structures (DNA, proteins, lipids, and organelles), leading to cell death in some cases [23].

Interestingly, oxidative stress induced by hypoxia exposure has been shown to cause endoplasmic reticulum dysfunction [24,25], which results in the accumulation of unfolded and/or misfolded proteins; this dysregulation contributes to the acceleration of several processes related to inflammatory pathways [26].

Several studies have shown a relationship between oxidative stress and hypobaric hypoxia exposure [8,10,14]. A recent study that exposed subjects to high altitude for 14 days showed an increase in capillary blood ROS production, which remained elevated from day 1 to day 14 [27]. In addition, oxidative biomarker analysis showed that the levels of protein carbonyls in the plasma increased from day 1 to day 7, those of urinary 8-hydroxy-2-deoxy guanosine (8-OH-dG) increased on the 2nd and 4th days, and the levels of urinary 8-isoprostane (8-iso-PGF2α) increased from the 1st day to the 4th day. In contrast, capillary antioxidant capacity decreased on the 1st, 2nd, and 4th days [27]. However, it is important to note that there are differences in oxidative and antioxidant responses among ethnic groups; for example, the Sherpa population in the Tibetan Plateau has a lower hemoglobin level and better oxygen saturation than lowlanders [28]. Additionally, a study by Horscroft et al. [29] showed that these individuals, compared to lowlanders, had a lower capacity for fatty acid oxidation and increased protection against oxidative stress in skeletal muscle. Moreover, a study by Julian et al. [30] found differences in antioxidant capacity (SOD and catalase) in Andean pregnancies compared to European pregnancies exposed to high-altitude conditions (3600–4100 m). Genetic differences in ethnic populations are intriguing and should be investigated in future studies.

Therefore, oxidative stress generated by increased ROS levels that is not compensated by antioxidant activity may contribute directly or indirectly to the development of pathologies associated with high-altitude exposure. Each of these high-altitude diseases will be separately discussed in the following sections.

## 3. Oxidative Stress and High-Altitude Diseases

### 3.1. Acute Mountain Sickness

Acute mountain sickness is the most common illness affecting unacclimatized people exposed to both acute [31] and intermittent [32] hypobaric hypoxia. The extent of this pathology mainly depends upon the individual’s speed of ascent and altitude [31]. The symptoms of AMS (headache, nausea/vomiting, fatigue and/or weakness, and dizziness/light headedness) can limit the activity and abilities of people experiencing this condition, especially during the first days of high-altitude exposure [33]. AMS can worsen, leading to HACE, which must be treated as a medical emergency [34]. However, HACE and AMS are currently considered different diseases even when patients present with both conditions at the same time [34].

Oxidative stress triggered by acute hypobaric hypoxia exposure damages several organs in the human body, especially the brain and heart [35]. Interestingly, a study demonstrated that AMS was correlated with an increase in the oxidative stress biomarker MDA, as measured in the breath [12]. Additionally, this study showed that oxidative stress can be a pathogenic factor for the development of AMS. This finding has been supported by several studies showing an increase in MDA concentration in several organs under hypobaric hypoxia conditions [12,14,36,37,38]. On the other hand, one study with subjects at 5100 m found no differences in the plasma MDA concentrations; however, subjects presenting with AMS showed increased lipid hydroperoxide levels and free-radical-induced vascular damage of the blood–brain barrier [39]. In addition, the use of lipid-soluble antioxidant vitamins was a potentially effective intervention that attenuated AMS [40]. Recently, a study with volunteers exposed to acute hypobaric hypoxia (4220 m; 36 h) found that all individuals presented with AMS after 8 h of exposure and showed increased MDA concentrations within 24 h of the start of the expedition [41].

However, in regard to antioxidant activity, a study by Tang et al. [38] (3500 m; 24 h) showed that 261 subjects had decreased SOD activity in plasma, suggesting that the decreased SOD activity was correlated with AMS severity. Additionally, a study by Irarrázaval et al. [41] showed glutathione peroxidase (GPx) activity decreased under hypobaric hypoxia conditions; this study emphasized the relationship between oxidative stress and AMS severity. Moreover, adults who had been born prematurely and were later exposed to hypobaric hypoxia (3840 m; 8 h) showed a decrease in antioxidant capacity and an increase in AMS severity [42].

In contrast, a proteomic analysis of plasma from subjects with AMS showed that the levels of oxidative-stress-protecting proteins, such as peroxiredoxin-6 (Prdx-6), GPx, and sulfhydryl oxidase 1 (QSOX1), were increased approximately twofold after 9 h of exposure to hypobaric hypoxia (4875 m), suggesting that the antioxidant activity of these proteins increases to regulate the high levels of ROS produced in response to hypobaric hypoxia [43]. These studies are summarized in Table 1. To clarify these contrasting results, more studies are necessary.

Regarding antioxidant treatment, a recent study with mice demonstrated that pretreatment with 5,6,7,8-tetrahydroxyflavone (5,6,7,8-THF), which has antioxidant properties, increased the activity levels of SOD, CAT, and GSH-Px and decreased the H_2_O_2_ and MDA contents in the brain and heart, indicating that the use of this flavone as an excellent antioxidant enhancer, lipid peroxidation inhibitor, and general anti-hypoxia agent for preventing AMS merits further exploration [35]. On the other hand, methazolamide, a lipophilic analog of acetazolamide with multiple advantages over acetazolamide that also functions as a carbonic anhydrase inhibitor to abolish AMS and HACE, has been considered an antioxidative stress drug [44] since a previous study showed that methazolamide can protect cultured neurocytes against H_2_O_2_-induced oxidative damage [45].

Additionally, because methazolamide can protect cerebral cortical neurons against oxidative stress and ameliorate cerebral-edema-inducing cases of AMS and HACE, researchers have proposed using methazolamide for the prevention and treatment of pathologies associated with high-altitude exposure [46]. These studies have shown that oxidative stress may be an important pathogenic factor in AMS physiopathology under acute hypobaric hypoxia, and that antioxidant treatment could be an important strategy to mitigate the severity of high-altitude sickness symptoms.

### 3.2. High-Altitude Cerebral Edema

High-altitude cerebral edema can develop from the progression of AMS, although these diseases are currently considered separate conditions [34]. HACE is a neurological disorder [47] characterized by signs and symptoms such as headache, loss of coordination, weakness, and impairments in consciousness [48,49]. Without appropriate treatment (such as reducing patient altitude), the individual may experience coma followed by death from brain herniation within 24 h [50].

Previous studies have reported that free radicals can damage endothelial cells of the blood–brain barrier, causing extracellular edema [51], which may contribute to the pathogenesis of both AMS and HACE [39,52]. To examine the roles of free radicals in these diseases, researchers have used sea buckthorn seed oil, which has antioxidant properties, to pretreat rats before exposure to acute hypobaric hypoxia (9144 m; 5 h) [13]. The results showed that this antioxidant pretreatment protected the brains of rats against transvascular leakage (altered cell membrane permeability) upon exposure to hypoxia by attenuating the generation of free radicals and MDA in the brain and increasing the antioxidant levels of GSH, GPx, and SOD [13]. This finding was later corroborated by a study showing that rats exposed to hypobaric hypoxia conditions (7620 m; 48 h) presented HACE with increased ROS and MDA levels and reduced antioxidant levels (SOD and GPx) in brain tissue [48]. The aforementioned increase in oxidative stress might upregulate the redox-sensitive transcription factor NF-κB, as indicated by studies showing an increase in NF-κB levels in the brains of rats exposed to acute hypobaric hypoxia [48,53]. In addition, NF-κB may contribute to transvascular leakage in the rat brain by upregulating the expression of pro-inflammatory cytokines (IL-6, IL-1, and TNF-α) and cell adhesion molecules (ICAM-1, VCAM-1, E-selectin, and P-selectin) [48].

Notably, treatment with ganglioside GM1, a neuroprotective sphingolipid, decreased the severity of HACE in rats exposed to acute hypobaric hypoxia (7600 m; 24 h), and the researchers of this study suggested that this neuroprotective effect was likely due to decreased ROS and MDA levels and increased SOD and GSH levels in rat brain tissue. Moreover, GM1 treatment decreased the expression of pro-inflammatory cytokines (IL-1β, TNF-α, and IL-6) in serum and brain tissue. In addition, GM1 activated the phosphoinositide 3-kinase (PI3K)/Akt-Nrf2 pathway, which decreases both oxidative stress and inflammation [54] (Table 1). Taken together, these studies indicate that oxidative stress induced by acute hypobaric hypoxia can contribute to HACE and that antioxidants are a promising treatment to mitigate HACE and AMS induced by hypoxia. However, to validate the specific antioxidant treatment that is most beneficial for this particular high-altitude pathology, more studies are needed.

### 3.3. High-Altitude Pulmonary Edema

High-altitude pulmonary edema is a non-cardiogenic, acute, and potentially lethal pulmonary disorder [10]. Rapid ascents to altitudes higher than 2450 m and exercise can lead to HAPE in unacclimatized individuals [47,55]. This illness usually manifests 2 to 3 days after acute exposure to high altitude and is characterized by increased pulmonary arterial pressure, hypoxic pulmonary vasoconstriction with elevated vascular permeability in alveolar capillaries, and hypoxemia [56,57].

Similar to that of HACE, the pathogenesis of HAPE in rats exposed to acute hypobaric hypoxia (7620 m; 24 h) involves increased ROS levels, trending toward levels causing oxidative stress and contributing to an increase in transvascular leakage; the same study showed that the expression of NF-κB, pro-inflammatory cytokines (IL-1, IL-6, and TNFα), and adhesion molecules (VCAM-1, ICAM1, P-selectin, and E-selectin) is increased, indicating that increases in ROS (H_2_O_2_), MDA, and pro-inflammatory molecule levels drive positive regulatory feedback [10]. Additionally, a recent study on HAPE induced by acute hypobaric hypoxia exposure (3250 m; 7 days) suggested that mutations in mitochondrial DNA complex I subunits may cause mitochondrial respiratory chain dysfunction, leading to oxidative stress, as determined by an increase in lipid peroxidation and protein carbonylation [58].

In addition, increased oxidative stress in HAPE patients compared to that in acclimatized controls may have been due to decreased SOD activity [58], although Sarada et al. [10] showed that after 6 h of exposure, the level of antioxidant enzymes, such as SOD, was increased in HAPE patients. This difference may be attributable to the duration of exposure to acute hypobaric hypoxia and the models used in the two studies mentioned in the paragraph above. Importantly, the levels of other antioxidants, such as GSH, did not differ, and those of GPx decreased [10] (Table 1). Therefore, we emphasize the importance of oxidative stress and the subsequent inflammatory response in HAPE pathology.

To attenuate oxidative stress, Purushothaman et al. [59] used sea buckthorn leaf extract as a pretreatment for HAPE in rats exposed to hypobaric hypoxia (9144 m; 5 h); this extract attenuated the generation of free radicals and MDA and increased GSH, glutathione reductase (GR), and GPx levels in the lung. Moreover, pretreatment with sea buckthorn leaf extract attenuated transvascular leakage into the lungs and decreased the expression of pro-inflammatory markers (TNF-α, IL-6, IL-10, and MCP-1) in bronchoalveolar lavage fluid [59]. Another study evaluating *Rhodiola crenulata* extract as a HAPE pretreatment—due to its antioxidant and ROS-scavenging properties—in rats exposed to hypobaric hypoxia (8000 m; 9 h) showed that this extract suppressed HAPE symptoms, possibly by reducing oxidative stress through decreased lipid peroxidation, H_2_O_2_, myeloperoxidase-H_2_O_2_-chloride system, and HIF-1α expression in the lung [60]. Additionally, this pretreatment reduced hypoxia-induced increases in plasma endothelin-1 (ET-1) levels and vascular endothelial growth factor (VEGF) levels in bronchoalveolar lavage fluid; both of these markers have been related to vascular leakage, and this increase was probably caused by inhibition of the oxidative stress-HIF-1 pathway [60]. Notably, studies in this section and throughout this review referring to the relationship between ROS (e.g., H_2_O_2_) and high-altitude sickness further support the role of oxidative stress.

### 3.4. Chronic Mountain Sickness

Chronic mountain sickness is a medical condition characterized by increased hematocrit levels and excessive polycythemia in patients living at high altitudes. This condition is also termed “Monge’s disease” because it was first described by Peruvian physician Monge [61]. CMS is typically characterized by at least three of the following symptoms: breathlessness, palpitations, sleep disturbance, cyanosis, vein dilatation, paresthesia, headache, or tinnitus, according to the Qinghai International Consensus scoring system [5]. This is a serious disease that is associated with morbidity and mortality [62]. In contrast to other pathologies associated with high altitude, CMS is found in residents living at high altitude (exposed to chronic hypobaric hypoxia), which is estimated to include 85 million people [63]; the prevalence of CMS (10–15%) can vary by altitude level, age, and genetic factors [5,64].

Interestingly, previous studies on residents at high altitude have shown that those with CMS present with higher oxidative stress markers (e.g., ascorbate radicals) and low antioxidant biomarkers (e.g., ascorbate) compared to residents at high altitude without CMS [65]. In addition, a recent CMS study by Bailey et al. [62] showed disequilibrium between free radical formation and antioxidant defenses in residents at high altitude, causing chronic systemic oxidative stress and inflammation. Moreover, this study showed that these oxidative and inflammatory responses are associated with accelerated cognitive decline and depression in residents at high altitude, particularly those with CMS [62].

In addition, 8-iso-PGF2α, an oxidative biomarker and a potent vasoconstrictor, is increased in people with CMS compared to people without CMS who live in the same high-altitude conditions. Furthermore, 8-iso-PGF2α is negatively associated with nocturnal saturation of oxygen and positively associated with hemoglobin concentration without alterations to SOD activity [66]. Moreover, a study on rats with CMS induced by chronic hypobaric hypoxia (5000 m; 30 days) demonstrated that in the CMS model group, the MDA level was significantly higher than that in the control group, and the concentrations of SOD and GPx were decreased [67]. Additionally, through a proteomic and metabolomics analysis, a recent study on rats with CMS induced by 28-day exposure to hypobaric hypoxia reported an increase in 8-hydroxyguanosine and trimethylamine N-oxide, which were associated with oxidative stress and pulmonary vasoconstriction processes, respectively [68] (Table 1).

Moreover, a study based on a proteomic analysis validated the potential plasma biomarkers in residents at high altitude with CMS, showing that hydrogen peroxide metabolic/catabolic, ROS metabolic, and acute inflammatory responses were significantly elevated. A protein–protein interaction analysis revealed that antioxidant activity, the hydrogen peroxide-induced catabolic process, and peroxidase and thioredoxin-1 activities constituted the key nodes in the molecular networks of CMS [8]. Therefore, it is important to consider increases in oxidative molecules (e.g., hydrogen peroxide) and oxidative biomarkers (e.g., MDA and 8-iso-PGF2α) and alterations to antioxidant activity (e.g., SOD and GSH-Px activity) as possible biomarkers for CMS in future studies.

### 3.5. High-Altitude Pulmonary Hypertension

One of the principal effects of hypobaric hypoxia exposure is hypoxic pulmonary artery vasoconstriction (HPV), which leads to the redistribution of blood to lung areas with the greatest ventilation [69]. Notably, when HPV is exacerbated and/or permanent, the pulmonary artery activates the vascular remodeling process, which increases vascular resistance and pulmonary artery pressure, leading to pulmonary hypertension—specifically HAPH [70,71,72].

Several studies have shown that increased ROS production induced by hypoxia is important to the development of HPV [73,74]. Interestingly, a recent study with rats under chronic hypobaric hypoxia (4200 m; 28 days) showed gradually decreased GPx and SOD activity on day 7 of high-altitude exposure; in contrast, the generation of both MDA and ROS increased in a time-dependent manner. Early high-altitude exposure first activates oxidative stress pathways; then, it gradually activates endoplasmic reticulum stress, which might promote apoptosis of alveolar epithelial cells.

The remodeling of pulmonary vessels has been correlated with the oxidative stress response during HAPH development [75]. This is supported by a study conducted by Bailey et al. [76] that exposed human subjects to acute hypobaric hypoxia (4559; 4–6 h) and found that the increases in pulmonary artery systolic pressure and vascular resistance observed at high altitude were associated with ROS molecules (ascorbate free radicals and lipid hydroperoxides), which altered endogenous vasodilator molecules, such as nitric oxide.

One of the principal consequences of HAPH is the development of right ventricular hypertrophy (RVH) due to excess pressure, which, in some cases, can trigger heart failure and ultimately lead to death [77]. Studies have also described the role of oxidative stress in the development of RVH under hypobaric hypoxia conditions, where it has been demonstrated that rats with RVH induced by exposure to chronic intermittent hypobaric hypoxia showed overexpression of NADPH oxidase complex-2 (Nox2) and p22phox and an increase in lipid peroxidation levels [37,78]. The link between RVH and oxidative stress is further supported by a study on rats under chronic hypobaric hypoxia conditions (5475 m; 28 days), which found that pulmonary artery vascular remodeling, RVH, and increased right ventricular systolic pressure occurred by the end of exposure; all of these effects were related to increased H_2_O_2_ and decreased SOD and GSH levels in the lung [79]. These results highlight the influence of oxidative stress on the pathological process of HAPH and the collateral cor pulmonale effects.

#### Pulmonary Artery Smooth Muscle Cell (PASMC) Remodeling

Several molecular alterations are involved in the development of pulmonary artery remodeling and subsequent HAPH, such as a decrease in the level of the endogenous vasodilator nitric oxide (NO) [6] and increases in asymmetric dimethyl arginine (ADMA) activity [80], intracellular calcium concentration ([Ca^2+^]_i_) [81], ET-1 expression [82], and IL-6 expression [83,84]; studies have demonstrated that all these molecules are related to oxidative stress, as discussed below.

The NADPH oxidase complex (specifically the Nox4 isoform) is considered to be a principal source of vascular pulmonary artery ROS production, which may be related to PASMC proliferation and pulmonary hypertension induced by hypobaric hypoxia [14]. NADPH is an electron donor complex that reduces molecular oxygen to superoxide (O_2_•^−^) or H_2_O_2_, depending on the isoform [85]; the predominant isoform in the pulmonary vascular system is Nox4, which has been shown to be overexpressed during pulmonary artery remodeling and in subsequent hypoxia-induced pulmonary artery hypertension (normobaric hypoxia; 10% O_2_; [86]). In particular, one study of chronic hypobaric hypoxia (4600 m; 30 days) reported an increase in the expression (mRNA and protein levels) of the Nox4 isoform and elevated levels of MDA in the lungs of rats with HAPH [14], findings corroborated by other studies [84,87]. A previous study on rats under chronic hypobaric hypoxia conditions (380 torr, four weeks) showed that the Nox2 isoform is necessary for HPV [88] (Table 1). Therefore, both Nox2 and Nox4 activity may directly or indirectly contribute to the development of HAPH induced by hypobaric hypoxia.

Additionally, given these findings, we hypothesize that the Nox2 isoform may be more closely related to the physiological HPV response and that Nox4 might contribute to the transition of HPV to PASMC remodeling and the development of subsequent pathology (HAPH) under hypobaric hypoxia. However, to confirm this hypothesis, further studies are needed.

NO bioavailability directly impacts PASMCs, inducing vasoconstriction and subsequent pulmonary hypertension [6]. The possible mechanism relating NO decrease to oxidative stress involves the reaction between superoxide and NO molecules that yields ONOO^−^, which is an oxidative molecule that contributes to exacerbated oxidative stress [6,14,86].

As previously mentioned, hypoxia-induced pulmonary hypertension is characterized by sustained pulmonary artery vasoconstriction and vascular remodeling, which are both mediated by PASMC contraction and proliferation. It is important to note that [Ca^2+^]_i_ is a major trigger for pulmonary vasoconstriction and an important stimulus for cell proliferation in PASMCs [89].

Studies have shown that oxidative stress or exacerbated levels of ROS (specifically superoxide and hydrogen peroxide) are necessary to increase [Ca^2+^]_i_ under hypoxic conditions; this concentration increase is realized through voltage-dependent calcium channels (VDCCs) in PASMCs [89,90,91] and causes PASMC contraction and increases in pulmonary artery pressure.

As mentioned above, several studies have determined that under hypoxic conditions, ET-1 is produced by various cell types in the vascular system, such as endothelial cells, macrophages, fibroblasts, and smooth muscle cells (SMCs) [92,93,94]. Studies have demonstrated that the increase in ET-1 expression may be associated with oxidative stress, increasing the proliferation of SMCs through activation of the NADPH oxidase complex [95,96].

Therefore, oxidative molecules (O_2_•^−^ and H_2_O_2_) and activation of NADPH oxidase (specifically the Nox4 isoform) may be related to altered expressions or concentrations of several molecules (Ca^2+^, NO, and ET-1) involved in the development of PASMC remodeling and subsequent HAPH.

Finally, we want to highlight the paucity of studies relating oxidative stress and high-altitude diseases that prompted us to conduct this review.

## 4. Conclusions

Exposure to high altitude (acute, chronic, and intermittent) increases the level of ROS induced by hypobaric hypoxia and typically decreases the effects of the antioxidant system, resulting in oxidative damage to several tissues and contributing to the development of several high-altitude illnesses.

The high-altitude diseases discussed in this updated review, such as AMS, HACE, and HAPE, are related to increased MDA levels and decreased SOD and GPx antioxidant activity. Additionally, oxidative stress contributes to the development of HACE and HAPE pathologies through the activation of inflammatory pathways induced by oxidative stress. Studies have shown an increase in MDA, 8-iso-PGF2α, and hydrogen peroxide levels and a decrease in SOD and GPx activity in CMS, all of which seem to be important biomarkers of this disease. Finally, in HAPH, the increase in Nox4 activity and ROS levels may contribute to the pulmonary artery remodeling process. Therefore, more studies on oxidative stress in high-altitude illnesses are necessary since ROS appear to be key to the development of high-altitude diseases. Antioxidant treatments could help to mitigate these high-altitude pathologies; however, since ROS molecules are related to physiological processes, these treatments should be carefully studied, and we urge caution when drawing conclusions about these treatments or extrapolating them to humans.

## Figures and Tables

**Table 1:** Oxidative stress and antioxidants in high-altitude diseases.

Pathology	Species	Hypoxic Condition	Oxidative Molecules and Biomarkers	Antioxidant Molecules	Sample	References
AMS	Human	Acute hypobaric hypoxia (3000–6125 m; 8 days)	↑ MDA		Breath	Araneda et al., 2005 [12]
	Human	Acute hypobaric hypoxia (5100 m; 20 days)	≈ MDA ↑ Lipid hydroperoxide		Plasma Serum	Bailey et al., 2001 [39]
	Human	Acute hypobaric hypoxia (4220 m; 36 h)	↑ MDA	↓ GPx	Plasma	Irarrázaval et al., 2017 [41]
	Human	Acute hypobaric hypoxia (3840 m; 8 h)	↑ MDA		Plasma	Debevec et al., 2020 [42]
	Human	Acute hypobaric hypoxia (4875 m; 9 h)		↑ Peroxiredoxin-6 ↑ GPx ↑ Sulfhydryl oxidase 1	Plasma	Julian et al., 2014 [43]
	Human	Acute hypobaric hypoxia (3500 m; 24 h)	↑ MDA	↓ SOD	Plasma	Tang et al., 2018 [38]
	Rat	Acute hypobaric hypoxia (7620 m; 24 h)	↑ ROS ↑ MDA ↑ Protein carbonylation	↓ Sulfhydryl content	Skeletal muscle	Agrawal et al., 2017 [36]
HACE	Rat	Acute hypobaric hypoxia (9144 m; 5 h)	↑ MDA	↓ GSH ↓ GPx ↓ SOD	Brain	Purushothaman et al., 2008 [13]
	Rat	Acute hypobaric hypoxia (7620 m; 48 h)	↑ ROS ↑ MDA	↓ GPx ↓ SOD	Brain	Himadri et al., 2010 [48]
	Rat	Acute hypobaric hypoxia (7600 m; 24 h)	↑ ROS ↑ MDA	↓ GSH ↓ SOD	Brain	Gong et al., 2018 [54]
HAPE	Human	Acute hypobaric hypoxia (3250 m; 7 days)	↑ MDA ↑ Protein carbonylation	↓ SOD ↓ GSH/GSSG	Plasma	Sharma et al., 2021 [58]
	Rat	Acute hypobaric hypoxia (7620 m; 24 h)	↑ ROS ↑ MDA	≈ GSH ↓ GPx ↑ SOD	Lung	Sarada et al., 2008 [10]
	Rat	Acute hypobaric hypoxia (9144 m; 5 h)	↑ MDA	↓ GSH ↓ GPx ↓ GR ≈ SOD	Lung	Purushothaman et al., 2011 [59]
	Rat	Acute hypobaric hypoxia (8000 m; 9 h)	↑ ROS ↑ MDA ↑ Myeloperoxidase		Lung	Lee et al., 2013 [60]
CMS	Human	Chronic hypobaric hypoxia (3600 m; high altitude residents)	↑ ROS ↑ Ascorbate radical	↓ Ascorbate	Plasma	Bailey et al., 2013 [65]
	Human	Chronic hypobaric hypoxia (3600 m; high altitude residents)	↑ Ascorbate radical	↓ GSH/GSSG	Plasma	Bailey et al., 2019 [62]
	Human	Chronic hypobaric hypoxia (4300 m; high altitude residents)	↑ 8-Iso-PGF2α	≈ SOD	Plasma	Julian et al., 2013 [66]
	Human	Chronic hypobaric hypoxia (4500 m; high altitude residents)	↑ H_2_O_2_ ↑ Peroxidase activity	↑ Thioredoxin-1	Plasma	Zhang et al., 2021 [8]
	Rat	Chronic hypobaric hypoxia (5000 m; 15 days)	↑ MDA	↓ GPx ↓ SOD	Plasma	Maimaitiyiming et al., 2014 [67]
	Rat	Chronic hypobaric hypoxia (5500 m; 4 weeks)	↑ 8-Hydroxyguanosine ↑ Trimethylamine N-oxide		Lung	Gao et al., 2020 [68]
HAPH	Human	Acute hypobaric hypoxia (4559 m; 4–6 h)	↑ Ascorbate free radical ↑ Lipid hydroperoxide		Plasma Serum	Bailey et al., 2010 [76]
	Rat	Chronic hypobaric hypoxia (4200 m; 28 days)	↑ ROS ↑ MDA	↓ GPx ↓ SOD	Lung	Pu et al., 2020 [75]
	Rat	Intermittent hypobaric hypoxia and chronic hypobaric hypoxia (4600 m; 30 days)	↑ Nox4 ↑ MDA		Lung	Lüneburg et al., 2016 [14]
	Rat	Intermittent hypobaric hypoxia and chronic hypobaric hypoxia (4600 m; 30 days)	↑ Superoxide ↑ p22phox		Pulmonary artery	Siques et al., 2014 [6]
	Rat	Intermittent hypobaric hypoxia (4600 m; 30 days)	↑ MDA ↑ Nox2 ↑ p22phox		Right ventricle hypertrophy	Pena et al., 2020 [37]

AMS, acute mountain sickness; MDA, malondialdehyde; ROS, reactive oxygen species; GPx, glutathione peroxidase; SOD, superoxide dismutase; GSH, glutathione; CAT, catalase; HACE, high-altitude cerebral edema; HAPE, high-altitude pulmonary edema; GSSG, glutathione disulfide; GR, glutathione reductase; CMS, chronic mountain sickness; HAPH, high-altitude pulmonary hypertension; RVH, right ventricle hypertrophy; Nox2, NADPH oxidase-2; Nox4, NADPH oxidase-4; ↑, increased level; ↓, decreased level; ≈, no changes.

## Abbreviations

AMS	Acute mountain sickness
CAT	Catalase
CMS	Chronic mountain sickness
ET-1	Endothelin-1
GPx	Glutathione peroxidase
GR	Glutathione reductase
GSH	Glutathione
H_2_O_2_	Hydrogen peroxide
HACE	High-altitude cerebral edema
HAPE	High-altitude pulmonary edema
HAPH	High-altitude pulmonary hypertension
HIF-1α	Hypoxia-inducible factor 1-alpha
HPV	Hypoxic pulmonary vasoconstriction
IL-1β	Interleukin-1β
IL-6	Interleukin-6
MCP-1	Monocyte chemoattractant protein-1
MDA	Malondialdehyde
NF-κB	Nuclear factor kappa B
NO	Nitric oxide
O_2_•^−^	Superoxide
•OH	Hydroxyl radical
ONOO^−^	Peroxynitrite
PASMCs	Pulmonary artery smooth muscle cells
PI3K	Phosphoinositide 3-kinase
ROS	Reactive oxygen species
RVH	Right ventricular hypertrophy
SMCs	Smooth muscle cells
SOD	Superoxide dismutase
TNF-α	Tumor necrosis factor-alpha
VEGF	Vascular endothelial growth factor
